# Informal caregivers’ perspectives on health of and (potentially inappropriate) medication for (relatively) independent oldest-old people – a qualitative interview study

**DOI:** 10.1186/s12877-018-0849-5

**Published:** 2018-07-25

**Authors:** Nadine Janis Pohontsch, Antje Löffler, Tobias Luck, Kathrin Heser, Debora Parker, Britta Haenisch, Steffi G. Riedel-Heller, Frank Jessen, Martin Scherer

**Affiliations:** 1 Department of General Practice / Primary Care, University Medical Center Hamburg-Eppendorf, Martinistr. 52, 20246 Hamburg, Germany; 2 Institute of Health Science, Brandenburg University of Technology (BTU) Cottbus-Senftenberg, Senftenberg, Germany; 3 Department of Economic and Social Sciences & Institute of Social Medicine, Rehabilitation Sciences and Healthcare Research (ISRV), University of Applied Sciences Nordhausen, Nordhausen, Germany; 4 Department of Neurodegenerative Diseases and Geriatric Psychiatry, University of Bonn, Bonn, Germany; 5 German Center for Neurodegenerative Diseases (DZNE), Bonn, Germany; 6 Center for Translational Medicine, University of Bonn, Bonn, Germany; 7 Federal Institute for Drugs and Medical Devices (BfArM), Bonn, Germany; 8 Institute of Social Medicine, Occupational Health and Public Health (ISAP), University of Leipzig, Leipzig, Germany; 9 Department of Psychiatry and Psychotherapy, University of Cologne, Cologne, Germany

**Keywords:** Potentially inappropriate medication, General practitioner, Informal caregiver

## Abstract

**Background:**

Oldest-old persons frequently receive potentially inappropriate medication. Medication use takes place under the patients’ informal caregivers’ influence. We explored informal caregivers’ perspectives on medication of (relatively) independent oldest-old persons to identify starting points for safer medication prescription/handling.

**Methods:**

In this exploratory qualitative interview study we interviewed 45 informal caregivers of 45 oldest-old persons (23 with potentially inappropriate medication/22 without potentially inappropriate medication). Interviews were recorded, transcribed and content analyzed (deductive/inductive coding).

**Results:**

Interviewees had little knowledge about/influence on oldest-old persons’ medication, but declared to monitor oldest-old persons’ needs for assistance. They were unaware of the concept of potentially inappropriate medication but sometimes sensitive to substance dependency. Most informal caregivers were satisfied with the oldest-old persons’ medication and viewed medication as increasing the patients’ quality of life. Inadequate communication was found between informal caregivers and general practitioners.

**Conclusions:**

Influence of informal caregivers on (relatively) independent oldest-old persons’ medication seems low. Stakeholders need to be aware that there is a transitional period where independency of oldest-old persons decreases and support needs increase which may be missed by (in-)formal caregivers or concealed by oldest-old persons. Monitoring patients’ medication competencies; measures supporting communication between informal caregivers and health care professionals; provision of educational and support resources for informal caregivers and the acceptance of oldest-old persons’ increasing assistance needs may increase medication safety.

## Background

Old-age individuals frequently receive potentially inappropriate medication (PIM), which is defined by an increased risk of causing harm due to side effects [1]. The German healthcare system uses the PRISCUS list (PL, [2]), which was published in 2010 listing individual drugs that are considered PIM. According to claims data, 25% of the individuals over the age of 65 receive at least one prescription for PRISCUS drugs in Germany per year [3]. This prevalence of PIM prescriptions is comparable to other European and non-European countries [4–6].

Adverse drug reactions (ADRs) are experienced by up to 30% of individuals over the age of 75 [7]. Particularly in elderly patients, the risk of hospitalization and death by PIM use increases up to 30% [1]. The estimated annual costs caused by PIM-related hospital admissions in Germany are 4 million EUR [8]. Long term use of PIM may also be linked to the increased risk of chronic diseases [9].

Usually, the patient- physician relationship and the treatment process are viewed as dyadic as long as the patient is still independent. Health behavior, use of health services and, therefore, also medication is known to take place under the influence of patients’ social groups, e.g., family, friends, and others [10–12]. The role of the people forming (relatively) independent patients’ social environments is often neglected. Different theories from the field of organizational and health psychology state the influence of subjective norms, peer pressure, and social environment on (health) intentions and behavior [12, 13]. Outside influence on medication use may increase with patients’ decreasing independency.

There are many studies on general practitioners’ (GPs, e.g., [14–16]) and patients’ [17–19] views on and role in PIM (de)prescription. Many studies have been carried out to get insight in informal caregivers’ role in the management of medication of dependent adults [20, 21] and older adults with dementia [22, 23] including a mixed studies review [24] showing that medication management is a very complex task burdening informal caregivers. One study was especially concerned about the impact of family caregivers on PIM in people with dementia [25].

Not all oldest-old people require help with their everyday needs [26]. Berlau and collegues [27] showed that there is a significant amount of people aged 90–94 years having no difficulties with activities of daily living. Data from the National Long-Term Care Survey shows that not all people from the older population (≥ 85 years) report disability concerning instrumental activities of daily living like grocery shopping or taking medication [28]. While those people undoubtedly may need some help from informal carers like relatives or friends they can be viewed as still living (relatively) independent. Dementia affects only approximately 20% of people aged ≥85 years [29] leaving a great proportion of this population unaffected. There is a significant lack of data from qualitative studies on the informal caregivers’ perspectives on relatively independent oldest-old adults without dementia regarding (long-term) prescriptions and use of PIM in current literature. The result is an insufficient understanding of the contextual framework where PIM is recurrently prescribed to oldest-old adults without dementia.

The current approaches to reduce PIM prescriptions have not been sufficiently successful, most likely because contextual causes beyond guidelines and warning list have not yet been fully identified and addressed. Thus, this study will focus on informal caregivers’ perspectives on health and (potentially inappropriate) medication of (relatively) independent oldest-old people not affected by dementia aiming at identifying starting points for deprescription and safer handling of PIM.

## Methods

This exploratory qualitative interview study was reviewed and approved by the following ethics committees: the Hamburg Medical Association (8 October 2014, MC-251/14), the University Hospital of Bonn (7 July 2014, 169/14) and the University of Leipzig (27 August 2014, 269–14-25,082,014). Interviews were conducted with GP patients’ [17], their informal caregivers’ and their GPs’ [14] from the AgeCoDe-Cohort (“German Study on Ageing, Cognition and Dementia in Primary Care Patients”, e.g., [30]) in Hamburg (HH), Bonn (BN) and Leipzig (L).

### Researcher characteristics

Interviews were conducted by AL, KH, and NJP who were employed on this project. Data was analyzed by AL and TL. AL is a female researcher (Master of Public Health; exemplary research areas: healthcare research, quantitative research). KH (exemplary research areas: neurodegenerative diseases, quantitative research) and NJP (exemplary research areas: potentially inadequate medication, general practice, quality indicators) are female postdoctorate researchers and trained psychologists. NJP has comprehensive experience in conducting semi-structured qualitative interviews and qualitative data analysis (for example [14, 31, 32]). KH is an experienced quantitative interviewer. AL and KH received training on qualitative interviewing and qualitative content analysis prior to data collection and analysis. TL is a male trained psychologist and full professor for Social Psychiatry (exemplary research areas: neurodegenerative diseases, quantitative research).

MS is a male full professor of Medicine, board certified in General Medicine. BH is a female specialized pharmacologist and university professor of Pharmacoepidemiology. SRH is a female full professor of Social Medicine and Public Health, MD board certified in psychiatry and psychotherapy and Master of Public Health. FJ is a male full professor of Psychiatry and Psychotherapy, board certified in psychiatry and psychotherapy. DP is a female biostatistician (Master of Applied Mathematics) and was working as a trainee at the time of the study.

### Participants and recruitment

We aimed at interviewing relatives (and others, i.e., partners and friends, in this manuscript referred to as ‘informal caregivers’) of GP patients taking PIM and those of GP patients corresponding in age and sex not taking PIM. The starting point of recruitment was the identification of eligible patients from the AgeCoDe-Cohort (for eligibility criteria see below). Following a snowball sampling approach [33] patients willing to be included in the study were asked to name an informal caregiver willing to be contacted by telephone to be invited to take part in the study. It is important to note that, although we refer to our interviewees as ‘informal caregivers’, most of the patients have not been dependent on the interviewees’ care and have been living independently or in assisted living facilities.

Eligible patients were defined by age ≥ 85 years, not having been diagnosed with dementia, and long-term use of PIM according to PL (see also [14, 17]). Every included patient taking PIM was complemented with another patient of the same sex and comparable age not taking PIM (non-PIM). PIM patients had been defined as having taken at least one PL drug since the last two available follow-up intervals. Non-PIM patients had not taken any drugs from PL in baseline or over any follow-up intervals. Eligible patients, who had consented to be contacted for other studies, were asked to participate in our study. We obtained the patients’ written informed consent to contact and interview their informal caregivers.

Inclusion criteria for informal caregivers were: 1) Potential interviewee must be a relative, partner or friend of an eligible patient and 2) the patient’s written consent for the informal caregiver to be interviewed. Exclusion criterion was the person’s unwillingness to be interviewed. All interviewees had given written, informed consent to be interviewed and for the interview to be digitally recorded, transcribed, and used for the study.

### Sample

All in all we included 52 patients [see also [14, 17]] and 45 informal caregivers (see Table 1; of 23 patients with PIM/22 patients without PIM, [14, 17]). Three patients were not able/willing to name an informal caregiver and four informal caregivers refused to take part in the study. The patients’ mean age was 89 years. Patients lived in different arrangements: alone in a private household (*N* = 20), private household together with spouse/partner (*N* = 7) or other relatives (*N* = 5), assisted accommodations (N = 7), nursing home (*N* = 3), alone in sheltered accommodation (N = 2), and in sheltered accommodation with spouse (*N* = 1).

**Table 1:** Interviewees relationship to oldest-old GP patients and level of involvement in medication management

Interviewees relationship to oldest-old patient		
	PIM (m/f)	NON-PIM (m/f)
Child	16 (9/7)	15 (5/10)
son-in-law	1 (1/0)	0
Wife/husband	3 (1/2)	2 (1/1)
Partner	1 (0/1)	0
Grandchild	1 (0/1)	2 (2/0)
Friend	0	2 (1/1)
Other relatives	1 (0/1)	1 (1/0)
Interviewees level of involvement in medication management		
	PIM	NON-PIM
Low involvement/support	12	16
Moderate involvement/support	8	4
High involvement/support	3	2

*PIM* Potentially Inappropriate Medication

Sixteen interviewees lived in and around HH, 17 in and around BN, and 15 in and around L. The interviewees lived either in the same household as the patients (*N* = 9), in different households in the same city (*N* = 23), or in different cities (*N* = 13). The level of involvement of interviewees in medication management was categorized into three groups based on qualitative interview data: low involvement/support (e.g., little knowledge about medication, no influence on medication management, no help with medication acquisition), moderate involvement/support (e.g., picking up prescriptions/medications at the doctors’ office/pharmacy, explicit knowledge about medications, discussing medications with the patient, application of injections) and strong involvement (e.g., controlling medication plans, preparation of daily doses, controlling intake). Many interviewees showed a low level of involvement (*N* = 28), while 12 interviewees were moderately involved, and five strongly involved (see Table 1).

Informal caregivers were interviewed face-to-face (*N* = 29) in their homes, the patients’ home, the study center, or by phone (*N* = 16). Sometimes other persons (e.g., the patient or a spouse) were present but not involved in the interviews. Interviews lasted Ø 34 min. PIM agent groups discussed included analgesics/anti-inflammatory drugs, antiarrhythmics, antibiotics, anticholinergics, antidepressants, antihypertensives/cardiovascular drugs, sedatives/hypnotic drugs and anti-dementia drugs/vasodilators/circulation-enhancing drugs (see also [14]).

### Interview guideline, data collection and transcription

The semi-structured interview guideline [34] was developed by AL, TL, SRH, and NJP. It was discussed and revised by the interdisciplinary CIM-TRIAD Study Group (areas of expertise represented: psychology, medicine, psychiatry, public health, and pharmacology). After piloting the guideline in three interviews with informal caregivers, minor adaptations were made. Interviewers introduced themselves stating their profession, affiliation, fields of expertise, role in the project, and experience before starting the interview. The interview guideline (see Table 2) consisted of questions concerning the patients’ medical history, their medications, the interviewees’ role concerning the medications, effects and side-effects of the medications, changes in medications, satisfaction with patients’ treatment and quality of life, interaction with the attending GP, and views on potentially problematic medications.

**Table 2:** Informal caregivers’ perspectives on health of and medication for oldest-old people – Interview guideline

I consult you today as a relative of XY. First of all, please tell me about the social environment you share.	
Please tell me about the medical history and medical treatment of XY.	
Please tell me everything you know about the medication XY is taking.	
Please describe, who prescribes these medications and how?	
Where does XY get his/her medication from?	
Which role do you play concerning XY’s medication intake?	
How do read up on XY’s medication and its positive and adverse effects?	
What do you expect from XY’s medication?	
Due to medication which positive effects does XY report?	
Due to medication which adverse effects does XY report?	
How does XY cope with adverse effects?	
How satisfied is XY with his/her medical treatment und medication effects?	
Please tell me about the effects of XY’s medication on his/her mood and activities of daily living.	
Please tell me to what extent XY and you were involved in the medical treatment.	
Are there any medications prescribed to XY that you consider problematic?	
In summary – How do you rate XY medical treatment overall?	
Do you want to add something? Are there any important aspects we didn’t talk about so far?	

Interviews using the interview guideline described above were conducted between December 2014 and July 2015 by AL (Master of Public Health), KH, and NJP (both trained psychologists / postdoctoral researchers). AL and NJP did not know any of the interviewees before the interview. KH knew six of the informal carers and had conducted quantitative interviews with two of them in the context of her previous work in the abovementioned AgeCoDe-study. Interviews were digitally recorded and transcribed by a trained research assistant using designated transcription rules and anonymizing all interviewees and patients. Transcripts were not returned to the participants. The transcripts’ accuracy was verified by AL, KH, and NJP.

### Data analysis

The transcripts were analyzed by AL and TL (trained psychologists/postdoctoral researcher) using structuring content analyses [35–37] following a realist approach [38]. The directed approach (inductive coding) and the conventional approach (deductive coding) were combined. Deductive codes were derived from existing literature (e.g., [20–22]) and the interview guideline. Categories were supplemented by inductive categories developed during the material reviews by AL in close consultation with TL. Main focus was placed on inductive category formation, as our study was of explanatory nature and ensured that the categories [35, 37] did not solely reflect pre-existing concepts. Deductive and inductive categories were described in code memos and supplemented with a descriptive example. Study participants did not provide feedback on the findings. To ensure intersubjective comprehensibility and credibility [39] of the analysis, the results were discussed in two face-to-face meetings of the CIM-TRIAD Study Group. Data was analyzed using MAXQDA 11 (Verbi GmbH).

## Results

Table 3 gives an overview of the main and subcategories described in the following sections.

**Table 3:** Overview of main and subcategories

Main category	Subcategory
Informal caregivers’ knowledge on and role in managing patients’ medication	• Knowledge of medical history • Monitoring of self-care capacities • Knowledge of prescribers and medications/information seeking • Familial roles • Potential to influence
Informal caregivers’ opinion on patients’ (potentially inappropriate) medication	• Increasing quality of life • Struggle to judge appropriateness • Satisfaction • Psychotropic drugs
Dyadic and triadic communication	• Lamenting • Reservation to share information • Discussing assistance needs • Communication with GPs

### Informal caregivers’ knowledge of and role in managing patients’ medication

Knowledge of patients’ health status was very diverse in the group of informal caregivers. While some could not tell anything about the patient’s health status, most informal caregivers knew about chronic diseases, past episodes of illness (e.g., cancer) or serious, acute diseases/events in the medical history. In many cases, illnesses and medications were not a predominant subject of discussion between the interviewed informal caregivers and the relatively independent patients. This had its roots in the informal caregivers’ subjective perceptions of an age-appropriate health status and independency level of the patients. As long as the patients were able to care for themselves, the informal caregivers tended not to interfere in the patients’ handling of illnesses and medication.

*“[…] I simply cannot, due to her fitness, I cannot constantly check on her, right? I just can’t do it.” [LCON02]*

Some informal caregivers showed sensibility for the fact that this status might change anytime. They reported to monitor the patients’ ability to handle medication and self-care and felt responsible to help or take over responsibility if necessary.

*“And the day will come, when I will have to deal with her health status. But for the time being, thank god, it is like that, that they both help themselves with the usual aids.* ”*[LPIM05]*

*“And I am also aware that something may happen very quickly, naah? But then I will go and get help immediately.” [HCON05]*

Many of the informal caregivers were not sure what types of medications the patients were taking. Some knew the type and purpose of medications taken, but most of the time they had no knowledge of exact agents or brand.

*“I don’t know the brand names. Altogether, she gets three medicines. The first one due to her weak heart, then diuretics and (…) oh well, the third one is one of those blood thinners. These are the three medicines but I don’t know their brand names.” [BPIM05]*

Symptoms like increased fatigue, decreasing physical strength, and restrictions in the ability to see and hear properly were often described as usual signs of aging. The same was true for decreasing social participation. Many informal caregivers reported increasing cognitive impairment of their patients. Usually, none of these symptoms were linked to the medications taken by the patients.

*“Well, actually my mother is healthy, she shows the normal signs of aging […] Anyway, she has resigned herself to the fact that she just simply does no longer participate in activities involving longer walking distances. But it is true, as far as we are able to understand it - it is painful for the afflicted – but surely a normal side effect of aging.” [LCON01]*

Informal caregivers had different approaches to informing themselves about the patients’ medication. Some searched actively for information, mainly using the package leaflets or the Internet, while others were not interested to do further research mainly because of regarding the medication responsibility to be the attending physician’s responsibility or being burdened with their own medication administration or health impairment.

*“So, for example, I have never read the patient information leaflets. Well, all that I have just told you is the knowledge that I have acquired by myself in the meantime in the sense that I myself also have to take different medications now. And (...) nope, basically, they have been prescribed over a longer period of time and nothing has changed and that’s why, at the time, I just simply took over and sorted them, I have to admit, without much concern. Not what they actually are for or against but that they have been prescribed and so, from my point of view, my responsibility was more for sorting them into the little [pill]box and that my mother takes them regularly.” [HPIM04]*

Informal caregivers were often unsure about the origin of the prescription. They often assumed that the GP must have been the prescriber. They also usually had no further knowledge about potential communication between different consulted physicians.

*“I think, it can only be the GP because of the high blood pressure”. [BCON01]*

Prescribed and over-the-counter medications were picked up from the pharmacy by informal caregivers if the patients were unable to pick up their medications themselves or they were resupplied by nursing services. Medication administration was supported by informal caregivers only if necessary.

*“She says: “I have to go to the doctor, I have to have new medicines”. Then I say: “I’ll call or I am going there anyway”. And then, (…), naah, I’ll do that. Well, the ordering, I’m doing all that now. She herself doesn’t do it anymore.” [BCON03]*

*“Well, she takes the medication, she knows what she has to take, she takes it on her own and she knows its effectiveness, side effects (laughs) and everything else, she is better informed at her age of [x years] as many others.” [BPIM07].*

In case of children-parent relationships, our interviewees reported different manifestations of parental and filial roles. Many patients were still relatively independent and insisted on managing their household and medication on their own. On one hand, this was a relief for their children but on the other hand, it did not allow to accept advice or help.

*“She says, don’t worry. I am only old. I am not sick. […] This is something I value very much about my mother that she always reassures me this way.” [LPIM05]*

*“Because, if you say something or ask something, then she replies: ‘I can still do all this by myself and as long as I am able to do this, it is no one else’s business.’” [BPIM06]*

There were cases where patients developed a fatalistic approach to life or lost the joy for life and therefore neglected self-care and medication adherence. Even the subjective potential exertion of influence of the informal caregivers was low.

*“Says she herself, she does not want to any longer. Honestly, I am a little concerned, if she gets worse, I remember the doctor in X-city then who said “Your mother doesn’t want to any longer.”, then I cannot influence her, when you don’t raise your desire to live. And this is, to some extent, the situation she always finds herself in. Where you constantly have to lift her spirits, sometimes even have to admonish her a little: “You have to get over it, you have to do something, you mustn’t hanker after it, not pity yourself.” Yes.” [HCON10]*

Other informal caregivers reported the increasing loss of independency of their patients. The informal caregivers are permitted to assume responsibility and thus gain influence on medication intake or adherence only if the patients accept their need for help. Sometimes this happens by organizing professional help.

*"[...] until recently, she really did everything on her own but she is no longer able to do it. She wanted to, for a long time she even did a little, it was difficult, to get that into her head. She didn’t want to accept it, I’ll just put it this way, that she was actually old now and that you have to accept help. It is difficult. For someone who actually lived totally independently, totally independently after my father’s death, to say now: “Naah, now you are ready for it.” [LPIM02]*

### Informal caregivers’ opinion about patients’ (potentially inappropriate) medication

Most informal caregivers considered the medication’s positive effects on the patients’ quality of life, e.g., absence of pain, to be very important. This was the most significant expectation reported. Positive effects were viewed as more important than possible negative effects. Most informal caregivers were not able to report subjectively perceived effects of any medications taken by the patients except in very serious cases. All in all, as little medication as possible was preferred for patients, while necessity of prescribed medications was rarely doubted.

*“No, actually, would prefer none, but she has to, hasn’t she? […]* ”*[BCON07]*

*“Personally I think, as little (medication) as possible with the best or most optimal effect.[…] *”*[LCON06]*

*“Oh well, but then again what are expectations, to me, that she is according to her age, well, (.) keeps her well-being (.) or her health but, well, she is [x years] now and (…) well, what am I to expect? I expect per se that she is pain-free or so. This is my main concern.” [HPIM06]*

*“And I have to say, well, it was the continuous intake of the medications and his attitude towards them that practically helped him to reach this age.” [LCON03]*

*“But this is positive; he no longer has any pain. (…) And he’d be stupid if he didn’t take a tablet now. (.) He’d shoot himself in the foot, then the pain returns. (.) Well, all pain medication is positive.” [HPIM07]*

All in all, informal caregivers were satisfied with the medical care the patients received. As they were generally medical laypeople, informal caregivers did not explicitly criticize the patients’ medication plans.

*“[…] But I think this medication is a good one. I cannot judge this by myself, but my sister told me, that this, this medication is very, very good. […]* ”*[BCON05]*

*“[…] I have difficulties to assess those medications […]*” *[HCON10]*

*“[…] I‘m too much of a layperson to overview that, I would say, if you change the medication now, medication X, whether she would be in a better mood or so. I would be a bad judge for that, no not bad, I cannot judge it at all. […]* ”*[HCON06]*

Though some critical voices raised concern about the potential addictiveness of some medications, e.g., benzodiazepines and opiates, but they fell silent by the desire for the patient’s well-being. Withdrawal was often not regarded an appropriate option, especially if the informal caregiver perceived the effects of the medication to be positive. The concept of PIM was not known to any of the informal caregivers.

*“Well, I see it this way: (.) that is now as layperson. I cannot assess the matter, I would say that it might somehow be a bit addictive / […], these are definitely opiates, they surely are somehow. But, I am not sure but it is like this: Without, she’d be poorly, right?” [BPIM02]*

*“[...] but you have to say, after fifty years it is no use anymore, to withdraw such an old woman from it now(.). You have to keep things in perspective, right? She isn’t sixty, she is [x years], right?” [HPIM02]*

Psychotropic drugs seemed to play a special role in the perception of informal caregivers. In our study, patients had often suffered from depression or anxiety for decades and those drugs had often been taken over a very long period of time (some of them were now PIM – either because of the patients’ old age or because of the long-term prescription). Even though the effects of those medications were often the only ones the informal caregiver could perceive and describe, i.e., they were very aware of them, the appropriateness of the intake was almost never questioned.

*“[…] Well, I know that she takes [brand name of the benzodiazepine]. I didn’t know at all how far back it dated. She just happened to mention to me that her GP back then, when she was 40 years old or still had been under 40, […]”[HPIM02]*

*“I know that my mother takes one medication to calm her. […] But when she doesn’t take them, she feels very poorly. (.) And here I also have to say, well, the tablet is very important, (…) because she isn’t even calm under normal circumstances.” [HPIM03]*

### Dyadic and triadic communication

Patients’ constant lamenting about their medical history, illnesses, and symptoms can function as attention-getter and method to strengthen (familial) ties. This may fuel the patient’s tendency to moan and demand attention which, in turn, may increase informal caregivers’ acceptance of (potentially inappropriate) medication intake because the patient seems to be suffering a lot. On the other hand, it may provoke informal caregivers’ resistance to care for the patient’s health status and may result in a refusal to follow up on the patients’ medication.

*“But so / this explains why we had been so close. […] Well, I also became a [profession] because of my mother, because I wanted to do everything better. That she somehow never is helpless. (.) And she evidently has taken advantage of it for decades. She has also always suffered from constant and severe migraines / (.) Oh well, and I always drove to her. Day and night. Whether I worked or not, didn’t I?” [HPIM02]*

*“She has always bragged about her medical history.” [HCON10]*

Some patients were described as very reserved in sharing information about their health status. Indicated reasons were: introversion, shame, privacy or general philosophy of life focusing on other life areas.

*“And we also didn’t talk about these things in our family and, naturally, this continued when old age arrived and we noticed that she, well, I am going to put this carefully now, in quotation marks, “typical pensioner” practicing doctor shopping, […]” [LPIM05]*

*“But then she is always so and, most of all, so fearful, naah? She doesn’t always like to say something and then she does everything and /. For example, also regarding this incontinence, it was you who pointed it out to me, I had no idea, did I? Where allegedly, the (…) doctor doesn’t prescribe this and so forth.” [HPIM02]*

Broaching the sensitive issue of (upcoming) assistance needs is sometimes viewed as paternalism and restriction of patient’s independency. Independent living was described as being very important for many patients and informal caregivers often did not interfere even with activities they did no longer approve of, e.g., driving a car while being visually impaired.

*“And apart from that I actually more or less (…) believe that it is important that she does as much as she is able to do on her own and I don’t, let me put it this way, continuously interfere in anything or take on different chores of hers where I believe that she is able to do all this by herself.” [HPIM08]*

*“And apart from that she still manages very well. She does everything on her own, unfortunately, she still drives her [brand name of the car] to go shopping and, yes / yes, also cares for [person X] who, however, really doesn’t want that at all, it is ‘n eternal conflict. But my mother regards it her mission, which she always (.) upholds, right? She / she says: ‚Nay, if I don’t have that, I won’t have anything to do.” [HPIM02]*

Communication between informal caregivers and GPs had almost never taken place because almost all patients had still been relatively independent. With increasing loss of independency, the informal caregivers became more and more involved in the (triadic) communication with GPs or communicated with them on their own, e.g., in case of hospitalization. Even then, prescriptions were rarely questioned.

*“She never goes to the doctor by herself. I always accompany her and this has also been agreed by Dr. X that I know about everything […]. We also have her living will. Doctor X knows this also, a power of attorney with all the bells and whistles.” [BCON07].*

*“And I made this clear to her, too, spoke again with Doctor X, and, after she had been discharged from the hospital, refrained from her having to go and see a pain therapist, as this actually also, and we also did this again, Doctor X and I in cooperation and also got it right and also had made ‘n plan.” [HCON02]*

*“Well, I would not dare to comment on this and say: “Yes, this is actually not necessary.” Well, I am not a doctor, the doctor prescribed it and surely must have had a few thoughts on the matter. At least that’s what I am thinking and now to say we urgently need something else, I just cannot permit myself to do that.” [HPIM04]*

## Discussion

### Summary of findings

We gained a considerable amount of information on informal caregivers’ knowledge and role in (relatively) independent oldest-old GP patients’ medication, their opinion on the patients’ (potentially inappropriate) medication and the communication processes between patients, informal caregivers and GPs. Our interviewees often had little knowledge about and influence on medication taken by the patients, but declared to monitor patients for assistance needs. They were unaware of the PIM concept but sometimes sensitive to possible substance dependency. Most informal caregivers were satisfied with the patients’ medication and viewed the medication as a means to increase the patients’ quality of life. Communication styles, such as lamenting or the reluctance to share information or discuss assistance needs were identified. Communication between informal caregivers and GPs was found to be inadequate.

### Strengths and weaknesses

To our knowledge, this has been the first study to focus on informal caregivers’ perspectives on the health of and (potentially inappropriate) medication for (relatively) independent oldest-old people not affected by dementia. We have therefore been able to gain important insights into what happens before an oldest-old person becomes increasingly dependent on care by (in-) formal caregivers and how this may be influenced by the person’s social environment. We had been able to recruit very different informal caregivers (family members and friends, male and female, younger and older people) and thus believe that we are able to provide a comprehensive view on the situation discussed. The level of interviewees’ involvement in medication management differed from low to high with more interviewees being less involved. This reflects the situation of the population of oldest-old patients as not every patient of oldest-old age is no longer able to care for themselves but has most often at least some support needs that might increase with progressing age [26–28].

### Findings relative to other studies

Different from people caring for dependent patients (with dementia) [20–22, 25], many interviewees did not play a decisive role in medication management and therefore often showed only fragmentary knowledge on health status, medication, and attending physicians. Some of the moderately and most of the strongly involved informal carers’ were able to give richer information about the patients’ health, attending physicians, and medication. Either way caregivers were unaware of the PIM concept. Informal caregivers’ information needs concerning PIM were also found in other contexts [25].

The interviewed informal caregivers reported a still relatively high degree of patients’ independency. Even so, some of the caregivers were already involved in medication management activities, such as organizing pill boxes or picking up medications from the pharmacy. We know from other studies that informal caregivers report struggles with medication management in relatively basic caregiving situations [20]. In most cases, one can assume that the independency of the oldest-old people will decrease with progressing age [40, 41]. The informal caregivers’ role in medication acquisition, intake, and monitoring usually also increases with increasing dependency of the oldest-old people [20, 21]. Most of the informal caregivers were medical laypersons struggling to decide on the medication’s appropriateness for the people in their care. They also reported to monitor their ‘dependents’ (possibly) declining health status or self-care abilities. We do not believe informal caregivers, as medical laypersons, should be responsible for determining the (potential in-) appropriateness of medication. However, they are adequately able to monitor and support the patients and health care professionals caring for them although there may be some training and information needs. (Self-) Medication competency of oldest-old patients needs to be regularly assessed by professionals [42] to determine when (additional) help is needed and informal caregivers need to be more involved in the care process.

As we are aware of the important role of informal caregivers in the healthcare for oldest-old people, especially for those who are developing cognitive impairment, we have to be sensitive to informal caregivers’ education, training and (psychosocial) support needs even in the very early stages of informal care [20, 43]. Further research has to be devoted to identify the information and support needs of (relatively) independent patients’ informal caregivers and whether their influence on medication provision, intake, and adherence can and needs to be increased in order to maximize medication safety.

Building rapport and effective communication strategies between general practitioners and (potential) informal caregivers of potentially increasingly dependent old persons must be facilitated early on without marginalizing patients. GPs have to motivate their elderly patients to incorporate potential informal caregivers early on, to discuss their health status and medication with them, and advise informal caregivers on medication handling and support and training possibilities. The inclusion of pharmacists into the network of carers to support informal carers and patients with ‘home-based’ medication reviews could be helpful [23] even for patients not affected by dementia. GPs need training in supporting informal caregivers in managing medications [24].

## Conclusions

Overall, we have seen the low influence of informal caregivers on (potentially inappropriate) medication for (relatively) independent oldest-old people. However, informal caregivers, health care professionals, and policy makers have to be made aware that there is a transitional period between the (relative) independency of oldest-old patients where support needs increase but may be missed by (in-)formal caregivers or covered up by the affected patients. Monitoring patients’ medication competencies, measures supporting communication between informal caregivers and health care professionals, provision of educational and support resources for informal caregivers as well as patients’ acceptance of increasingly needing help can improve medication safety in this substantial patient population.

## Abbreviations

ADR: Adverse drug reaction
AgeCoDe: ‘German Study on Ageing, Cognition and Dementia in Primary Care Patients’
BN: Bonn
CIM-TRIAD: ‘Contextual background for chronic use of inappropriate medication at high age: A qualitative study with physicians, patients and relatives’
GP: General practitioner
HH: Hamburg
L: Leipzig
PIM: Potentially inadequate medication
PL: PRISCUS list
